# Cost-effectiveness of semaglutide versus dulaglutide for Type 2 Diabetes in China: A Markov Model analysis

**DOI:** 10.1371/journal.pone.0351059

**Published:** 2026-06-10

**Authors:** Qiying Chen, Tianyu Chen, Weicheng Lin, Xi Chen

**Affiliations:** 1 Department of Pharmacy, The Second Affiliated Hospital of Fujian Medical University, Quanzhou, China; 2 Department of Nursing, Quanzhou Medical College, Quanzhou China; Hospital Infantil de México Federico Gomez: Hospital Infantil de Mexico Federico Gomez, MEXICO

## Abstract

**Objective:**

From the perspective of China’s basic medical insurance, to evaluate the cost-effectiveness of semaglutide versus dulaglutide for type 2 diabetes mellitus (T2DM) in China, informing clinical and health policy decisions.

**Methods:**

This study employed a Markov model to simulate the disease progression of T2DM over a 25-year time horizon. Transition probabilities between health states were sourced from the UK Prospective Diabetes Study Outcomes Model (UKPDS 82) and the SUSTAIN 7 clinical trial, while health utility values were obtained from published literature using the Chinese EQ-5D-5L value set. Long-term cost-effectiveness was projected through cohort simulation, with both one-way sensitivity analysis and probabilistic sensitivity analysis (PSA) conducted to evaluate the robustness of the model.

**Results:**

After 25 years of simulation, the total cost for dulaglutide was ¥193,353.07, with 11.628 quality-adjusted life-years (QALYs) gained. The total cost for semaglutide was ¥240,925.08, yielding 11.900 QALYs. Compared with dulaglutide, semaglutide had an incremental cost-effectiveness ratio (ICER) of ¥174,904 per QALY gained, approximately twice China’s 2023 per capita GDP (¥89,358). According to the Chinese Guidelines for Pharmacoeconomic Evaluation (2020), this result falls within the economically acceptable range (1 × GDP < ICER ≤ 3 × GDP). One-way sensitivity analysis showed that although health state utility values were the most influential parameter, the model results remained robust. PSA indicated that at a willingness-to-pay (WTP) threshold of 3 times the per capita GDP (¥268,074), semaglutide had an 89.6% probability of being considered economically acceptable.

**Conclusion:**

From the perspective of China’s basic medical insurance, semaglutide demonstrates cost-acceptable outcomes compared to dulaglutide in managing type 2 diabetes. This analysis provides crucial evidence for clinical decision-making and health policy formulation.

## Introduction

As living standards rise, the convergence of excessive caloric intake, declining physical activity, and population ageing is driving a sharp increase in diabetes mellitus in China, and T2DM now accounts for 90–95% of all cases [1]. These factors, coupled with the chronic nature of the disease and its high burden of complications, will markedly amplify healthcare expenditures in China. National cross-sectional surveys (2013–2018) document a rise in adult diabetes prevalence from 10.9% to 12.4%, while pre-diabetes climbed from 35.7% to 38.1% [2]. A Markov projection based on identical demographic assumptions forecasts that prevalence among Chinese adults aged 20–79 years will increase from 8.2% in 2020 to 9.7% by 2030, with total diabetes-related health-care spending surging from US$250.2 billion to US$460.4 billion [3]. These converging epidemiological and economic indicators establish T2DM as a foremost public-health priority in China.

Current glucose-lowering therapies targeting pancreatic β-cells are limited by adverse events, notably hypoglycaemia and weight gain. Glucagon-like peptide-1 receptor agonists (GLP-1RAs) overcome these limitations via glucose-dependent insulin secretion, glucagon suppression, and delayed gastric emptying, resulting in a low risk of hypoglycaemia. Both domestic and international guidelines now recommend GLP-1RAs for T2DM management [4,5]. Structural modifications—specifically fatty acid acylation and albumin-binding motifs—markedly prolong their half-life, enabling once-weekly subcutaneous administration. This regimen effectively sustains glycated hemoglobin (HbA1c) targets and improves adherence [6].

The 2020 Chinese Guidelines for the Prevention and Control of Type 2 Diabetes advise adding GLP-1RAs with proven cardiovascular benefit to metformin-based therapy in confirmed T2DM [7]. Nonetheless, data from the Chinese cohort of the multinational CAPTURE study (n ≈ 9,800 across 13 countries) revealed an overall cardiovascular disease prevalence of 33.9% among T2DM patients, with atherosclerotic cardiovascular disease (ASCVD) accounting for 94.9% of these cases [8]. Strikingly, only 1.5% of Chinese T2DM patients with comorbid ASCVD received GLP-1RAs [8], despite clear guideline recommendations [7,9], underscoring a substantial evidence-to-practice gap. These findings underscore that as T2DM management becomes increasingly standardized in China, the utilization of GLP-1 RAs is anticipated to rise substantially. As the growing number of these agents enter the Chinese market, establishing appropriate pricing strategies for healthcare policymakers and identifying the most cost-effective options for clinicians have become pressing needs.

Semaglutide (approved in China on 14 August 2021) and dulaglutide (approved in China on 16 June 2019) are once-weekly GLP-1RAs with well-established clinical efficacy and safety profiles [5]. However, systematic pharmacoeconomic evaluations of their long-term use remain limited within China’s healthcare cost framework. Furthermore, reliable long-term real-world cost data are still scarce due to their relatively recent introduction to the Chinese market. To address this evidence gap, we developed a Markov state-transition model that integrates international clinical trial data with contemporary Chinese parameters to evaluate the lifetime cost-effectiveness of semaglutide versus dulaglutide in managing T2DM. From the perspective of China’s basic medical insurance, another key objective of this study was to establish a streamlined pharmacoeconomic modeling framework for T2DM that supports rapid parameter substitution, enabling efficient generation of economic evaluations for different glucose-lowering agents. This framework aims to provide evidence-based guidance for clinical selection of GLP-1RAs and facilitate optimal allocation of healthcare resources in China. Transparency and reproducibility of this study were ensured through strict adherence to the Consolidated Health Economic Evaluation Reporting Standards 2022 (CHEERS 2022) [10] (see S1 Table) throughout both the design and reporting stages.

## Materials and methods

### 1. Data sources

#### 1.1. Markov Model.

**Fig 1:**
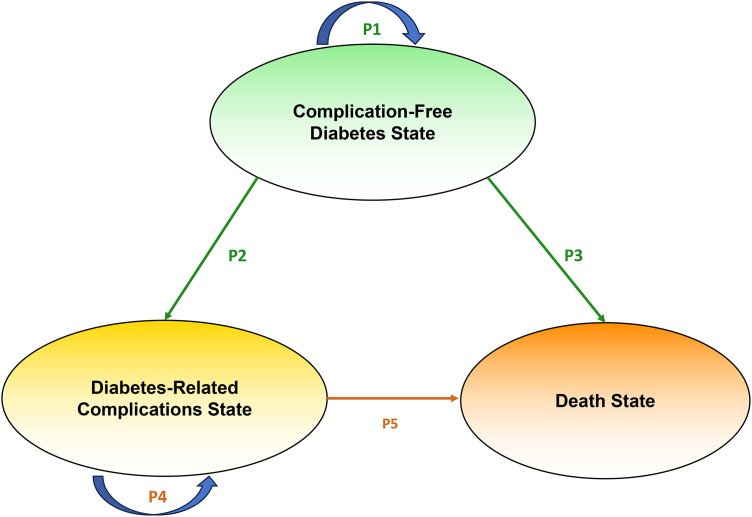
State transition diagram of the Markov model for T2DM.

Markov modeling is well-suited for simulating chronic diseases as it can simultaneously capture long-term costs and health outcomes [11]. Our initial model structure included three distinct health states: diabetes without complications, multiple diabetes-related complications (macrovascular and microvascular), and death (an absorbing state). However, further disaggregation into a dozen or more complication-specific states substantially increased model complexity and computational burden. To balance computational feasibility with the available data, previous domestic studies consolidated all complication states into a single “diabetes with complications” state, resulting in a parsimonious three-state model: “diabetes without complications,” “diabetes with complications,” and “death” [12–16]. This reduced the number of states from over ten to three, significantly improving computational efficiency (Fig 1). In the diabetes disease progression model, patients may exist in one of three health states: uncomplicated state, complicated state, or death state. From the uncomplicated state, patients may remain in the current state (probability = P₁), progress to the complicated state (probability = P₂), or transition to the death state (probability = P₃). From the complicated state, patients may remain in that state (probability = P₄) or progress to death (probability = P₅). The death state serves as an absorbing state from which no reversible transitions to other states occur. Consistent with the natural history of diabetes, reverse transition from the complicated state to the uncomplicated state is not permitted.

Acknowledging that the aggregation of complications into a single state may sacrifice granularity and potentially introduce structural bias, we adopted the three-state framework but implemented a key refinement to enhance its clinical plausibility. Specifically, both the disease state transition probabilities and health utility values for the aggregated “diabetes with complications” state were not uniform but were derived as a prevalence-weighted average of the probabilities for major complication subtypes. This design incorporated the following considerations:

- Target of Weighting: The weighting was primarily applied to the composite calculation of complications.
- Source of Weights: The weights were based on the real-world prevalence profile of macrovascular (68%), microvascular (19%), and co-existing complications (13%), as reported by the China National Diabetes and Metabolic Disorders Study (CNDMDS) 2023 [17].
- Intended Effect: This approach ensures that the model’s disease progression is more heavily influenced by the more prevalent complication types, thereby more realistically reflecting the heterogeneous impact of distinct complications.
- Underlying Logic: The use of cross-sectional prevalence data serves as a proxy for the long-term relative risk of developing different complications within the cohort.

This weighting strategy mitigates the risk of oversimplification inherent in a pure three-state model, while preserving computational transparency and efficiency. The specific calculation formula and its application to health state utilities and other relevant parameters are detailed in the subsequent section.

T2DM is a chronic metabolic disorder characterized by a prolonged course and the delayed onset of complications, which typically emerge years after the initial diagnosis. To simulate this protracted progression, a Markov model with 1-year cycle lengths was developed. The model cohort’s starting age was set at 50 years, reflecting the mean age at T2DM diagnosis in China [18]. A time horizon of 25 years was adopted to simulate disease progression until patients reached the age of 75. This horizon captures the patient’s “core burden of disease” period, covering the years where the risks of diabetes-related complications and associated costs are highest. This approach is further justified by evidence indicating that the life expectancy of Chinese patients with T2DM is often shorter than the general population life expectancy of 77.93 years [19], making an endpoint at age 75 clinically relevant and sufficient to capture the long-term economic and clinical outcomes of interest. All patients entered the model in the ‘diabetes without complications’ health state. Owing to the extended time horizon, we rigorously applied discounting protocols specified in the Chinese Guidelines for Pharmacoeconomic Evaluations (2020 Edition) [20]: we applied a 5% annual discount rate to both costs and health outcomes. Additionally, per guideline recommendations, sensitivity analyses explored discount rate variations (0%–8%) to quantify parameter uncertainty. It should be noted that in the present study, we assumed that semaglutide and dulaglutide were used as monotherapies continuously over the 25-year simulation period of the Markov model, and real-world medication behaviors such as treatment switching and drug discontinuation were not incorporated. After constructing the Markov model using TreeAge Pro (TreeAge Software, Williamstown, MA), we conducted sequential analyses: rollback computation, Markov cohort simulation, Monte Carlo simulation, and sensitivity analysis.

#### 1.2. Cost Parameters.

From the perspective of China’s basic medical insurance, treatment costs in pharmacoeconomic evaluations typically encompass direct costs, indirect costs, and intangible costs. However, this perspective naturally restricts the cost scope to direct medical costs—since China’s basic medical insurance primarily covers direct medical expenses such as drugs, diagnostic tests, and hospitalization, and does not include indirect costs (e.g., productivity losses) or intangible costs. Accordingly, consistent with prior studies by Chinese scholars [15–16], this study limited the evaluation exclusively to direct medical costs, which include:

(1) Drug costs—Semaglutide (2023 National Reimbursement Drug List (NRDL)-negotiated price: ¥813.96/4 mg specification/ pen, maintenance dose 1 mg/week, 4-week supply per vial) and dulaglutide (2023 NRDL price: ¥123.55/1.5 mg specification/vial, maintenance dose 1.5 mg/week) were evaluated using larger pack sizes，consistent with long-term protocols in Chinese Type 2 Diabetes Mellitus Prevention Guidelines 2020.
(2) Routine management costs—Derived from the CNDMDS 2023 national Diagnosis-Related Group (DRG) database [17], including basic treatment: ¥3,860/year.
(3) Complication treatment costs—From same source [17]: with macrovascular and microvascular events averaged ¥19,720/year.
(4) Adverse event costs—Excluded because GLP-1 RA-related gastrointestinal symptoms are self-limited (medical intervention rate:1.2%−3.5% [21]), and discontinuation rates were below significance thresholds in the SUSTAIN-7 trial [22]（dulaglutide 2.4%, semaglutide 3.1%). Full cost structures in Table 1 (2023 values).

**Table 1:** Cost information of the two treatment strategies.

Drug	State	Drug Cost (¥)	Background Treatment Cost (¥)	Complication Treatment Cost (¥)	Total Cost (¥)
**Semaglutide**	**Complication-free**	10 548.92	3 860.00	——	14 408.92
	**With complications**	10 548.92	3 860.00	19 720.00	34 128.92
**Dulaglutide**	**Complication-free**	6242.60	3 860.00	——	10 284.60
	**With complications**	6424.60	3 860.00	19 720.00	30 004.60

#### 1.3. Health Utilities and Transition Probabilities.

Health-state utilities for QALY calculations were sourced from published literature [23–27]. The utility value for diabetes without complications was not estimated by discounting perfect health (1.00) but was directly derived from the population norm reported by Liu et al. [25]. The EQ-5D-5L utility values used in their study originated from a cross-sectional survey of urban Chinese adults (≥18 years; N ≈ 1,296) conducted across five cities in China [24]. Participants first described their health status using the EQ-5D-5L descriptive system, then self-rated it on the EQ-VAS; responses were converted to a 0–1 utility index using the Chinese EQ-5D-5L value set. After age-weighting to the 30–59-year stratum (peak diabetes incidence range), the utility was 0.88, which we adopted as the baseline for the no-complications state.

Health utility values for the with-complications states were obtained from a cross-sectional study involving 2,202 Chinese community-dwelling adults with type 2 diabetes conducted by Huang et al. [28]. The utility values for different complication states, adjusted for demographic characteristics, are presented in Table 2.

**Table 2:** Health State Utility Values for T2DM with Complications.

Complication Type	Utility Value (Mean ± SD)	References
**Macrovascular events**	0.735 ± 0.108	[25,28]
**Microvascular events**	0.791 ± 0.101	[25,28]
**Both macrovascular and microvascular events**	0.701 ± 0.115	[25,28]

Based on the complication profile from the CNDMDS 2023 (macrovascular: 68%; microvascular: 19%; co-existing: 13%), a weighted average utility for the composite “diabetes with complications” state was calculated using the formula: (0.68 × 0.735) + (0.19 × 0.791) + (0.13 × 0.701) = 0.74. This computation integrates the respective utility values for each complication subtype, weighted by their real-world prevalence, to derive a more representative composite utility. The utility for the death state was defined as 0.00. These utility values are consistent with the well-established reference ranges commonly used in technology appraisals by the National Institute for Health and Care Excellence (NICE; https://www.nice.org.uk).

A key parameter in this study was primarily derived from the SUSTAIN 7 trial [22], a multinational, randomized, open-label, phase 3b study designed for the head-to-head comparison of once-weekly semaglutide versus dulaglutide. The trial enrolled adults (≥18 years) with type 2 diabetes mellitus who had inadequate glycemic control on metformin-based therapy, with baseline HbA1c levels ranging from 7.0% to 10.5%. Glycemic outcome data—specifically the change in HbA1c from baseline over the 40-week treatment period—were extracted from this trial. The head-to-head design of SUSTAIN 7 enabled a direct comparison of the two interventions, minimizing biases inherent in indirect treatment comparisons and thereby providing reliable input parameters for the cost-effectiveness analysis. The incidence rates for diabetes-related complications were obtained from the UKPDS82 [29], a landmark cohort study that provides well-validated estimates of complication probabilities. The state we generated the transition parameters for the Markov model by integrating the HbA1c changes observed for semaglutide and dulaglutide in the SUSTAIN 7 trial with the complication probabilities from the UKPDS82. The use of these established data sources enhances the credibility and predictive validity of the long-term outcome projections in this study.

Glycemic outcome data from the SUSTAIN 7 trial—specifically the changes in HbA1c, systolic blood pressure (SBP), and body weight were used to derive the annual transition probabilities between health states in the Markov model. The relative treatment effect of semaglutide versus dulaglutide was estimated by applying the HbA1c, SBP, and body weight reduction differences to established risk equations [29], which were then integrated into the model’s transition matrix to simulate long-term clinical and economic outcomes.

The Changes in HbA1c serve as a core efficacy measure for all glucose-lowering medications, with data being readily accessible and officially validated. Therefore, this study established differentiated mapping relationships between HbA1c reductions and corresponding decreases in complication risks according to complication type. For microvascular complications, we estimated the risk reduction at approximately 37% (range: 33%−41%). Based on this, a 10% relative reduction in HbA1c was estimated to correspond to a risk reduction of approximately 35%−41%. A Beta distribution was adopted to reflect the uncertainty around this estimate, as this distribution is commonly used in probabilistic sensitivity analysis to model uncertain parameters bounded between 0 and 1. Regarding macrovascular complications, the benefits of early intensive glucose-lowering remain uncertain and have not reached statistical significance. Therefore, we used a conservative uniform distribution to represent the potential range of risk reduction, as this distribution reasonably reflects the possible interval of risk reduction effect in the absence of precise data. Annual transition probabilities were derived from event rates (r) using the declining exponential approximation of life expectancy (DEALE) formula [30]:


$$\begin{array}{c} P=\text{1}-exp(-rt) \end{array}$$

While HbA1c serves as a validated surrogate endpoint for glucose-lowering medications and a well-established predictor of microvascular complications, the progression of T2DM and the development of its complications are driven by multiple interdependent risk factors. These include, but are not limited to, systolic blood pressure, lipid profiles (e.g., LDL-C), body weight, smoking status, and renal function. In recognition of this multifactorial pathophysiology, our modeling strategy does not attribute complication risks solely to changes in HbA1c. Instead, it leverages the UKPDS82 dataset [29], which inherently incorporates the combined effects of these risk factors as observed longitudinally in a well-characterized cohort, within this analytical framework, HbA1c reductions observed in the SUSTAIN 7 trial [22] function as a primary, though not exclusive, determinant of microvascular risk, and as one of several contributors to macrovascular risk, against the background of the integrated risk profile emulated by the UKPDS structure. This design ensures that long-term projections reflect a clinically plausible and holistic representation of disease progression, rather than a reductionist, glucose-centric approximation. Adjusted annual event rates were subsequently converted into state transition probabilities (Table 3).

**Table 3:** Annual incidence rates and transition probabilities between T2DM states.

State	Item	Drug						References
		Metformin		Dulaglutide		Semaglutide		
		Incidence Rate	Transition Probability	Incidence Rate	Transition Probability	Incidence Rate	Transition Probability	
**Complication-Free Diabetes State**	**Complication-free (P1)**	——	0.9659	———	0.9707	———	0.9786	[22,29,30]
	**With complications (P2)**	0.0277	0.0273	0.0238	0.0235	0.0173	0.0172	[22,29,30]
	**Death State (P3)**	0.0068	0.0068	0.0058	0.0058	0.0043	0.0042	[22,29,30]
**Diabetes-Related Complications State**	**With complications (P4)**	——	0.9933	——	0.9943	——	0.9958	[22,29,30]
	**Death State (P5)**	0.0067	0.0067	0.0058	0.0057	0.0042	0.0042	[22,29,30]

#### 1.4. Willingness-to-Pay (WTP) Threshold.

Consistent with China’s 2020 Guidelines for Pharmacoeconomic Evaluation, this study established per-capita gross domestic product (GDP) as the WTP benchmark, implementing a three-tiered decision rule wherein ICER below one times GDP are considered cost-effective, those between one and three times GDP are deemed acceptable, and values exceeding three times GDP are regarded economically unsustainable [17,18]. Based on China’s 2023 per-capita GDP of ¥89,358 (National Bureau of Statistics [31]), we set the threshold (λ) at three times this value (¥268,074), defining the maximum acceptable incremental cost for the intervention. This specific base year was selected because all cost inputs in the model—including drug acquisition costs and medical expenditures—were sourced from 2023. Aligning the WTP threshold with the same base year as the cost data ensures temporal consistency across all model parameters and avoids potential bias that could arise from using GDP figures from different years. This practice is consistent with the methodological recommendations of the China Guidelines for Pharmacoeconomic Evaluations, which advise that the threshold year should correspond to the year of the cost data.

#### 1.5. Sensitivity Analysis.

Given the inherent limitations of pharmacoeconomic models and uncertainties in key parameters, study findings may not fully reflect real-world outcomes. We therefore conducted systematic sensitivity analyses to examine result robustness, comprising: One-way sensitivity analysis: Individual variation of critical parameters to quantify their isolated impact on outcomes; Probabilistic sensitivity analysis: Simultaneous perturbation of all relevant parameters according to their probability distributions (e.g., β for probabilities, γ for costs) to evaluate overall model uncertainty.

## Results

### 1.1. Cost-Effectiveness Analysis

A Markov cohort model was developed to compare the long-term cost-effectiveness of semaglutide versus dulaglutide in Chinese patients with type 2 diabetes mellitus. Acknowledging that conventional Markov models assume constant transition probabilities while complication risks increase exponentially with age and disease duration. Within TreeAge Pro software, half-cycle corrections were applied to costs, health utilities, and other relevant parameters to address potential bias from the extended cycle length. To more strictly adhere to international guidelines recommending lifelong time horizons, we additionally incorporated a 35-year scenario analysis (simulating patients up to age 85 years) based on the original model to evaluate the impact of an extended time horizon on the ICER. Over a 25-year time horizon, semaglutide was associated with higher total costs (¥240,925.08 vs. ¥193,353.07) and greater QALYs (11.900 vs. 11.628) compared with dulaglutide, resulting in an ICER of ¥174,904 per QALY gained. When the time horizon was extended to 35 years, semaglutide yielded an ICER of ¥142,481 per QALY, with incremental costs of ¥53,897.41 and incremental QALYs of 0.378. Both ICERs fell China’s 2023 willingness-to-pay threshold of 3 × GDP per capita (¥268,074/QALY). Notably, the ICER decreased with longer time horizon, suggesting improved cost-effectiveness over time. Comprehensive results are presented in Table 4.

**Table 4:** Cost-effectiveness analysis results of the two treatment regimens.

Time Horizon	Treatment Regimen	Cost (¥)	Effect (QALYs)	Incremental Cost (¥)	Incremental Effect (QALYs)	Cost-Effectiveness (¥/QALY)	ICER (¥/QALY)
**25-year**	Dulaglutide	193 353.07	11.628	——	——	16 628	——
	Semaglutide	24 0925.08	11.900	47 572.00	0.272	20 245	174 904
**35-year**	Dulaglutide	233 259.21	13.222	——	——	17 642	——
	Semaglutide	287 156.61	13.600	53 897.41	0.378	21 114	142 481

### 1.2. Results of Markov Cohort Analysis for Two Antidiabetic Treatment Regimens

Markov cohort simulations at cycle 25 revealed divergent health-state distributions between the treatments. Compared to dulaglutide, semaglutide was associated with a lower proportion of patients in the death state (9.99% vs. 13.80%), a higher proportion in the complication-free state (58.23% vs. 47.54%), and a lower proportion in the complicated state (31.79% vs. 38.97%). These differences, derived from the model projections, were stable across PSA.

### 1.4. One-Way Sensitivity Analysis

**Fig 2:**
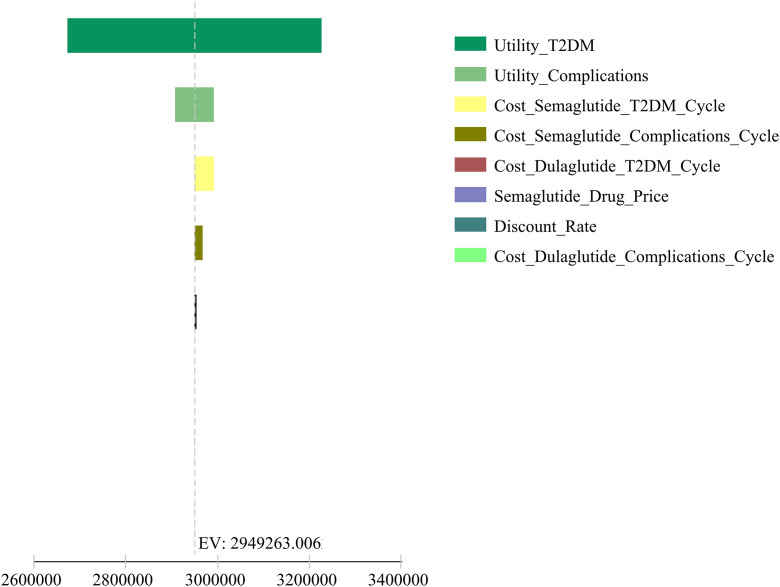
Tornado analysis (Nte Benefits).

This study conducted one-way sensitivity analyses on costs between health states, health-state utilities, the discount rate, and the unit price of semaglutide for the two treatment regimens. Parameters were varied as follows: Reflecting China’s recent implementation of Diagnosis-Related Groups/Diagnosis-Intervention Packet (DRG/DIP) and payer cost-containment objectives, the costs for both the semaglutide and dulaglutide strategies were reduced by 25% from baseline values. Recognizing that semaglutide—despite price negotiations reducing its cost from the initial launch price of ¥1,904.00 to ¥813.96 per pen—remains a higher-priced newer agent with greater potential for further price reduction compared to the earlier-marketed dulaglutide, its unit price was further reduced 20% reduction from baseline values. Health-state utilities were varied by ±10% from baseline. The discount rate was tested across a range of 0% to 8%. The tornado plot confirms that health utility values exert the strongest influence on net benefit, while markedly narrower sensitivity bands for other parameters underscore structural stability. Results remain robust across plausible ranges for most inputs. Critically, the baseline net benefit decisively exceeds the WTP threshold, creating a safety margin. Even extreme-but-plausible utility variations maintain net benefit well above the threshold, effectively eliminating reversal risk from parameter perturbations. These analytical findings indicate that the model’s conclusions are robust to parameter uncertainty (Fig 2).

### 1.5. Probabilistic Sensitivity Analysis

**Fig 3:**
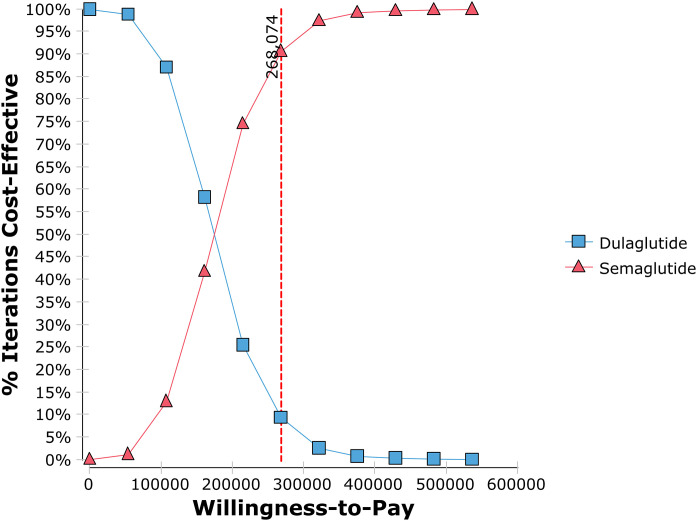
Cost-Effectiveness Acceptability Curve.

**Fig 4:**
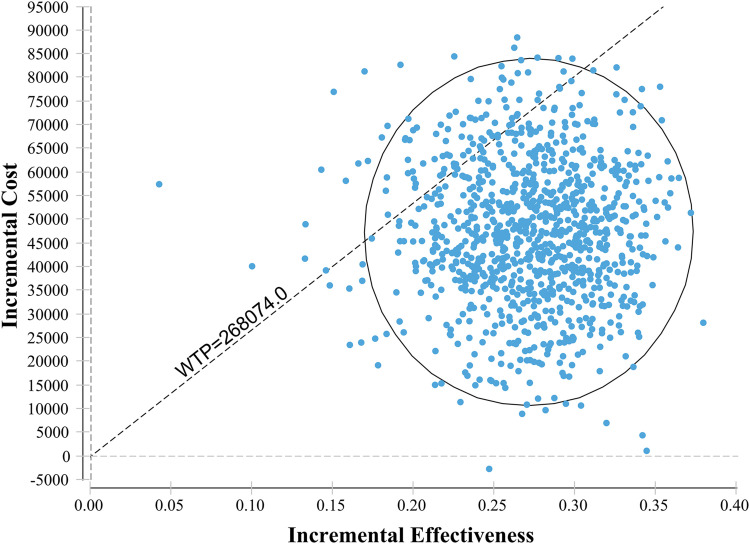
Incremental Cost-Effectiveness, Semaglutide v. Dulaglutide.

To assess the robustness of the cost-effectiveness findings, we conducted a PSA incorporating uncertainty across all model parameters. We assigned parameter distributions based on their characteristics: Gamma distributions for costs, Beta distributions for health utility values, and Normal distributions for the discount rate. The mean for each distribution was set to its baseline value. The standard deviation was then derived using the formula (Upper limit – Lower limit)/ (2 × 1.96) [15,16]. Full distributional specifications are detailed in Table 5. We performed a Monte Carlo simulation with 1,000 iterations. Results were visualized through scatter plots of incremental costs and effectiveness (semaglutide vs. dulaglutide) and cost-effectiveness acceptability curves (CEACs). At the baseline WTP threshold of ¥268,074 per QALY, the PSA revealed that the majority of ICERs for semaglutide versus dulaglutide fell below this threshold (Fig 3). Consistent with this, the CEAC demonstrated that semaglutide had a 89.6% probability of being cost-effective compared to dulaglutide at this WTP threshold (Fig 4). These PSA results support the economic acceptability of semaglutide versus dulaglutide at the specified WTP threshold.

**Table 5:** Model input parameters and distributions.

Probability Sensitivity Parameter	Distribution Type	Mean	Standard Deviation	References
**Semaglutide cost (complication-free) (¥)**	Gamma	14 470.55	922.87	[15–17]
**Semaglutide cost (with complications) (¥)**	Gamma	34 190.55	2 180.52	[15–17]
**Dulaglutide cost (complication-free) (¥)**	Gamma	10 291.82	656.37	[15–17]
**Dulaglutide cost (with complications) (¥)**	Gamma	30 011.82	1 914.02	[15–17]
**Semaglutide unit price (¥)**	Gamma	813.96	41.53	
**Health utility value (complication-free)**	Beta	0.88	0.05	[23–27]
**Health utility value (with complications)**	Beta	0.74	0.04	[25,28]
**Discount rate**	Normal	0.05	0.02	[20]

## Discussion

In 2017, China formally integrated pharmacoeconomic evaluation reports into the core evidence for National Healthcare Security Administration price negotiations. The 2019 policy directive mandating that “comparisons among similar drugs follow pharmacoeconomic principles” further solidified the discipline’s pivotal role in reimbursement decisions. Nevertheless, pharmacoeconomic research in China started later than in high-income countries. Systematic literature reviews confirm a persistent scarcity of real-world data specific to Chinese T2DM patients. Consequently, the UKPDS remains the primary source for epidemiological and utility inputs in most diabetes-related economic evaluations in China [32]. Expanding China-specific real-world evidence is therefore essential for advancing pharmacoeconomics domestically [33]. Pharmacoeconomics quantifies pharmaceutical “value” through systematic, scientific comparison of economic costs and health outcomes across therapeutic alternatives. This informs healthcare decisions, optimizes resource allocation, and maximizes population health [34]. High-quality pharmacoeconomic studies are critical for generating, synthesizing, and appraising evidence to guide decisions and promote rational drug use [35]. Such evidence now explicitly informs the NRDL dynamic adjustment process, particularly regarding the inclusion and pricing of high-cost innovative medicines [36].

We employed a modeling approach to complement the limitations of real-world studies. These two paradigms differ fundamentally in evidence-based decision-making, with distinct methodological orientations and evidence generation logics. Modeling studies extrapolate from the best available evidence to predict potential outcomes of different intervention strategies under idealized, long-term scenarios, with strengths lying in their prospective nature and efficiency. In contrast, real-world research focuses on actual clinical practice that has already occurred, reflecting the effectiveness and safety of treatments in real-world settings through observational data, with core value derived from its practical relevance and external validity. We employed a Markov model in this study primarily to overcome the inherent limitations of traditional randomized controlled trials regarding follow-up duration and sample size. The SUSTAIN-7 trial lasted only 40 weeks, whereas diabetes, as a chronic progressive disease, requires long-term observation to fully manifest the economic and health impacts of its complications. We used discrete-time simulation to extrapolate short-term clinical indicators (such as the reduction in glycated hemoglobin) to a lifetime horizon. This allowed us to simultaneously estimate long-term endpoints including cumulative complication incidence, mortality, and quality-adjusted life years. Furthermore, the model translates clinical endpoints into a common health economics metric—the incremental cost-effectiveness ratio—providing an intuitive and quantifiable basis for resource allocation decisions. Our framework supports rapid head-to-head comparisons of different intervention strategies and systematically assesses the impact of parameter uncertainty on conclusions through probabilistic sensitivity analysis, significantly enhancing the robustness of the results. Leveraging the established model structure allows for quick adjustments of drug types and related parameters to obtain simulation outcomes. Achieving such lifetime perspective inferences solely through real-world research would be exceptionally challenging.

This study has several methodological limitations that merit attention. First, while most parameters (including drug costs and health utility values) were obtained from Chinese data sources, the disease transition probabilities primarily rely on epidemiological data from Western populations (e.g., UKPDS). Potential differences in disease characteristics between Chinese and Western populations should be considered when interpreting these findings. Second, the model utilizes a conventional Markov structure with static transition probabilities. Although half-cycle corrections were applied to costs, health utilities, and other relevant parameters to mitigate bias from the extended cycle length, this framework fails to fully capture the dynamic evolution of risks with disease progression or treatment adjustments. Additionally, the model assumed constant transition probabilities over the 25-year horizon, which may not fully capture the time-varying nature of complication risks as patients age and disease progresses, potentially introducing minor biases in long-term complication rate estimations. Furthermore, the cost analysis was limited to direct medical costs, excluding indirect and intangible costs that constitute important components of the total economic burden. A third limitation involves the modeling of treatment effects. Although based on the multifactorial UKPDS risk equations, the intervention effect was predominantly driven by between-group differences in HbA1c reduction. This approach, while methodologically standard, may overemphasize glycemic control as the primary mechanism for long-term risk reduction—particularly for macrovascular outcomes where non-glycemic factors (e.g., blood pressure, lipids) play crucial roles. Although most GLP-1RAs have demonstrated pleiotropic benefits on various cardiometabolic parameters (including body weight and cardiovascular risk), the current model structure could not explicitly quantify the independent contributions of these non-glycemic effects due to limited availability of integrated long-term evidence. Notably, our model was based mainly on glycemic and macrovascular endpoints derived from the SUSTAIN-7 trial, without explicitly incorporating emerging pleiotropic cardiovascular benefits, particularly the protective effect of semaglutide against atrial fibrillation (AF). Recent meta-analyses have indicated that semaglutide significantly reduces AF risk [37], while the REWIND trial showed no AF benefit for dulaglutide [38], and a separate meta-analysis even suggested a potential increased risk [39]. Given the lack of direct head-to-head comparative data on AF outcomes between the two agents, these potential differential cardiovascular effects were not captured in our model, which may make the incremental economic value of semaglutide somewhat conservative. Future studies should integrate more comprehensive cardiovascular endpoints to refine long-term economic evaluations. Fourth, this study assumed continuous use of semaglutide or dulaglutide over the entire 25-year simulation period, representing an ideal treatment pathway. In real-world clinical practice, treatment discontinuation, switching, or escalation to basal insulin after one year—as documented in Chinese real-world studies (Liu et al. [40]; Hu et al. [41]; Ruan et al. [42])—may reduce the long-term effectiveness of both agents. This idealization may overestimate the long-term benefits of both agents. Future research should incorporate real-world treatment patterns to develop more complex treatment pathway models. Despite these limitations, comprehensive sensitivity analyses confirm that the main findings remain robust across plausible parameter ranges. Thus, this study provides valuable preliminary evidence regarding the economic profile of GLP-1RAs in China. Future research should prioritize developing Chinese-specific diabetes models through: (1) establishing longitudinal real-world databases to enhance parameter localization; (2) creating more accurate individualized risk prediction models; (3) performing comprehensive economic evaluations that include indirect and intangible costs; and (4) strengthening structural uncertainty analysis to ensure independent effects of HbA1c, systolic blood pressure, lipids, and body weight on complication risks are adequately accounted for. These improvements would allow more precise estimation of the long-term value of contemporary glucose-lowering therapies.

T2DM is an incurable chronic metabolic disease characterized by progressive microvascular and macrovascular complications [43–45]. Lifelong continuous treatment is required, and inappropriate treatment switching may lead to adverse clinical outcomes [8]. Although both semaglutide and dulaglutide are once-weekly GLP-1RAs, existing evidence indicates that semaglutide holds clinical advantages in several aspects: the SUSTAIN-7 trial demonstrated its superiority over dulaglutide in reducing HbA1c and body weight [22]; a recent randomized trial published in *Diabetes, Obesity and Metabolism* reported less injection site pain with semaglutide [46]; semaglutide allows flexible dose adjustment, whereas dulaglutide requires a fixed-dose, single-use auto-injector [47]; semaglutide has a half-life of approximately 7 days, which helps maintain stable plasma drug concentrations, compared to approximately 5 days for dulaglutide [48,49]. Additionally, in China, semaglutide has approved indications for cardiovascular risk reduction and renal protection that dulaglutide does not, while dulaglutide has an approved pediatric indication not available for semaglutide [48,49]. Based on this, the present study further evaluated its cost-effectiveness by constructing a Markov model for long-term analysis. Over a 25-year simulation, the total cost for the dulaglutide regimen was ¥193,353.07, yielding 11.628 QALYs; the total cost for the semaglutide regimen was ¥240,925.08, yielding 11.900 QALYs. The ICER for semaglutide compared to dulaglutide was ¥174,904 per QALY gained, which falls within the economically acceptable range in China. Both one-way sensitivity analysis and probabilistic sensitivity analysis demonstrated robust model results. The modest incremental health gain of 0.272 QALYs accurately reflects the inherent pharmacological similarity between the two GLP-1RAs and the modest but statistically significant clinical differences observed in the SUSTAIN-7 head-to-head trial. Notably, in long-term lifetime cost-utility analyses for chronic metabolic diseases, even modest incremental QALY gains can translate to meaningful population-level benefits when extended over a lifelong horizon and applied to large patient populations—particularly in China, which has the world’s largest T2DM burden. Therefore, from the perspective of China’s basic medical insurance, semaglutide shows certain advantages over dulaglutide in the management of T2DM, providing important evidence for clinical medication decisions and health policy formulation.

Our findings align with and extend prior Chinese pharmacoeconomic research. Consistent with Hu et al. [41], we confirm that semaglutide offers superior cost-effectiveness over dulaglutide in China. However, our study advances this evidence by (1) employing a long-term continuous treatment design to quantify the theoretical upper bound of GLP-1RA benefits; (2) integrating synergistic effects of glycemic, weight, and blood pressure reductions over a 25-year horizon; and (3) generating China-specific ICERs using local parameters. This continuous treatment design, while differing from real-world modeling approaches such as Risebrough et al. [49] which incorporated treatment switching to reflect clinical practice, is methodologically aligned with international standards for assessing theoretical drug value—both approaches are valid within the same Markov modeling framework, each serving distinct research objectives. Relative to Ruan et al. [42], which focused on short-term (24–52 week) outcomes, our Markov modeling captures long-term complication risk reductions and QALY gains. Furthermore, we incorporate systolic blood pressure improvements—a dimension absent in Ruan et al. [42]—to more comprehensively reflect the pleiotropic benefits of GLP-1RAs. Compared to the systematic review by Wang et al. [50], which synthesized global evidence on GLP-1RA efficacy and real-world cost-effectiveness, our study translates those findings into localized economic data, demonstrating that semaglutide’s ICER (¥174,904/QALY) falls well below China’s three-times-GDP threshold, thereby confirming its cost-effectiveness advantage specifically for Chinese T2DM patients.

## Conclusion

This study, based on long-term Markov model simulations, demonstrates that although the direct drug cost of semaglutide is higher than that of dulaglutide, from a long-term health economics perspective, the health outcome improvements brought by this treatment regimen result in an incremental cost-effectiveness ratio that falls within China’s WTP threshold, indicating that semaglutide is cost-acceptable relative to dulaglutide. The findings not only provide reliable pharmacoeconomic evidence for the dynamic adjustment of the NRDL and the optimization of clinical pathways but also offer a scientific basis for promoting outcome-oriented precision medication practices. These results hold significant reference value for enhancing the glycemic control rate, quality of life, and long-term health outcomes of patients with type 2 diabetes in China. Future studies incorporating real-world treatment patterns and Chinese-specific epidemiological data will further strengthen the generalizability of these findings.

## Supporting information

S1 Table CHEERS 2022 Checklist for Health Economic Evaluations. (DOCX)
