# Supplementary material for: Cost-effectiveness of semaglutide versus dulaglutide for Type 2 Diabetes in China: A Markov Model analysis
Source: PLoS One. 2026 Jun 10;21(6):e0351059. doi: 10.1371/journal.pone.0351059 (PMC13252742; doi:10.1371/journal.pone.0351059)
Supplement: S1 Table — (DOCX) [file pone.0351059.s001.docx]

This study was reported in accordance with the Consolidated Health Economic Evaluation Reporting Standards (CHEERS) 2022. The compliance of each reporting item and the corresponding explanations of this study are presented as follows:

**Supporting information 1. CHEERS 2022 Checklist for Health Economic Evaluations**

| **No.** | **Reporting Item** | **Compliance (√/Partially compliant/×)** | **Explanations of This Study** |
| --- | --- | --- | --- |
| 1 | Title and Abstract: Clearly state the type of economic evaluation (cost-effectiveness/cost-utility/cost-benefit analysis) and the study perspective | √ | The title and abstract explicitly indicate a cost-effectiveness analysis, and clarify the study perspective as the *perspective of the Chinese basic medical insurance payer*. |
| 2 | Research Question: Clearly describe the interventions, comparators, and study population | √ | The study aims to evaluate the cost-effectiveness of semaglutide 1.0 mg once weekly versus dulaglutide 1.5 mg once weekly, with the study population being patients with T2DM consistent with the inclusion criteria of the SUSTAIN-7 trial. |
| 3 | Research Objective: State the core objective of the evaluation (e.g., compare cost-effectiveness to provide evidence for decision-making) | √ | The core objective is to quantify the ICER of the two GLP-1 RAs, so as to provide evidence-based support for medical insurance policy-making and clinical rational drug use in China. |
| 4 | Target Population: Clarify the baseline characteristics, inclusion and exclusion criteria of the study population | √ | The baseline characteristics of the study population are strictly based on those of T2DM patients in the SUSTAIN-7 head-to-head trial, with clear and specific inclusion and exclusion criteria defined. |
| 5 | Interventions: Detail the dosage regimen and treatment course of the intervention and comparator | √ | The administration frequency and dosage of the two drugs are clearly specified; the simulation course of the model is set to 25 years, in line with the natural course of T2DM as a chronic progressive disease. |
| 6 | Study Perspective: Clarify the perspective and the rationale for its selection, and list the scope of costs/benefits under this perspective | √ | The perspective of the Chinese basic medical insurance payer is adopted, with the rationale of meeting the actual needs of domestic medical insurance fund management and policy-making in China. Only direct medical costs borne by the medical insurance payer are included in the cost calculation. |
| 7 | Study Time Horizon: Clarify the model simulation time horizon and the rationale for its selection | √ | The simulation time horizon is 25 years. This setting is based on the natural course of chronic progressive T2DM, the general design of similar long-term pharmacoeconomic studies in China, and the actual clinical context that the average onset age of T2DM is 50 years old and the life expectancy is approximately 75 years old in the Chinese population. |
| 8 | Discount Rate: Clarify the discount rate for costs and health outcomes, and state the basis for the setting | √ | The discount rate is set at 5%, which strictly complies with the optional range of discount rates specified in the *Chinese Guidelines for Pharmacoeconomic Evaluation (2020 Edition)*. |
| 9 | Cost Estimation: Clarify the cost type, estimation method and data source, and list the specific cost values | √ | Only direct medical costs are estimated, including drug costs, diagnosis and treatment costs, and complication treatment costs. The data are derived from the bidding prices of drugs and the charging standards of public medical institutions in China. |
| 10 | Health Outcomes: Clarify the health outcome indicators (QALY), and state the measurement method and source of utility values | √ | The quality-adjusted life year (QALY) is used as the core health outcome indicator. Utility values are based on the Chinese norm of the EQ-5D scale and calibrated according to different T2DM disease states (without complications/with complications). |
| 11 | Model Selection: Clarify the adopted economic model and describe the model structure and state classification | √ | A three-state Markov model is adopted, with disease states classified as *T2DM without complications, T2DM with complications, and death*. The transition probabilities of each state are calculated based on the UKPDS 82 risk equation and the SUSTAIN-7 trial. |
| 12 | Model Parameters: List all core parameters (efficacy, transition probability, cost, utility value) and their data sources | √ | All core parameters are clearly marked with their data sources, including the UKPDS 82 risk equation, SUSTAIN-7 trial, Chinese Guidelines for the Diagnosis and Treatment of Diabetes, and classic domestic pharmacoeconomic studies. References for each transition probability parameter are supplemented in Table 2 and Table 4. |
| 13 | Sensitivity Analysis: Describe the type (one-way/probabilistic/scenario), method and results of sensitivity analysis | √ | Comprehensive sensitivity analyses are conducted, including one-way sensitivity analysis, probabilistic sensitivity analysis (Monte Carlo simulation with 1000 iterations), and scenario analysis. The detailed results and robustness verification of all analyses are reported in the main text. |
| 14 | Incremental Analysis: Clearly report incremental costs, incremental health outcomes, and incremental cost-effectiveness ratio (ICER) | √ | The core results focus on the ICER of semaglutide versus dulaglutide. The cost-effectiveness is judged by comparing the ICER with the commonly used threshold of pharmacoeconomic evaluation in China (3 times the per capita GDP of China). |
| 15 | Uncertainty Analysis: Explain the uncertainties in the model and their potential impacts on the results | √ | The potential uncertainties caused by model assumptions (e.g., constant transition probabilities over 25 years, continuous medication without treatment switching) and their impacts on the study results are clearly explained in the Limitations section. |
| 16 | Study Limitations: Objectively elaborate on the limitations of study design, data and model, and propose future research directions | √ | An independent section of Limitations is set to detail the deficiencies in transition probability setting, treatment path design and population generalization, and specific improvement directions for future research are proposed (e.g., constructing a time-varying transition probability model combined with real-world data). |
| 17 | Study Conclusions: Provide targeted decision-making recommendations based on the study results | √ | Specific and operable decision-making recommendations for clinical drug selection and medical insurance fund allocation are proposed based on the ICER results and the actual situation of T2DM treatment in China. |

**Note**: All items in this study are **fully compliant (√)** with the CHEERS 2022 reporting standards, with no partially compliant or non-compliant items.

**Abbreviations**: GLP-1 RA, glucagon-like peptide-1 receptor agonist; T2DM, type 2 diabetes mellitus; QALY, quality-adjusted life year; ICER, incremental cost-effectiveness ratio; GDP, gross domestic product; UKPDS 82, United Kingdom Prospective Diabetes Study Outcomes Model.
